# Supplementary material for: Improved Placement of Multi-mapping Small RNAs
Source: G3 (Bethesda). 2016 May 11;6(7):2103–11. doi: 10.1534/g3.116.030452 (PMC4938663; doi:10.1534/g3.116.030452)
Supplement: Supplemental Material [file supp_6_7_2103__index.html]

Improved Placement of Multi-mapping Small RNAs — Supplemental Material 

# Improved Placement of Multi-mapping Small RNAs

## Supplemental Material for Johnson *et al.*, 2016

**Files in this Data Supplement:**

- File S1 - *sRNA-simulator.py*: Python script used to create simulated sRNA-seq datasets. (.txt, 67 KB)
- Table S1 - Dataset accession numbers and descriptions. (.xlsx, 97 KB)
- Table S2 - Versions and settings. (.xlsx, 78 KB)
- Table S3 - Oligo sequences. (.xlsx, 291 KB)
- Table S4 - Methods used for placement of MMAP sRNA-seq reads in 20 previous studies. (.xlsx, 72KB)
